# Ironing out complexities in karst chronology: (U-Th)/He ferricrete ages reveal wet MIS 5c

**DOI:** 10.1126/sciadv.adp0414

**Published:** 2024-10-02

**Authors:** Matej Lipar, Milo Barham, Martin Danišík, Andrej Šmuc, John A. Webb, Kenneth J. McNamara, Aleš Šoster, Mateja Ferk

**Affiliations:** ^1^ Anton Melik Geographical Institute, Research Centre of the Slovenian Academy of Sciences and Arts, Ljubljana 1000, Slovenia.; ^2^ Timescales of Mineral Systems Group, School of Earth and Planetary Sciences, Curtin University, Perth 6845, Australia.; ^3^ Western Australia ThermoChronology Hub (WATCH) Facility, John de Laeter Centre, Curtin University, Perth 6845, Australia.; ^4^ Department of Geology, Faculty of Natural Sciences and Engineering, University of Ljubljana, Ljubljana 1000, Slovenia.; ^5^ Discipline of Ecology and Environment, La Trobe University, Melbourne 3086, Australia.; ^6^ School of Earth Sciences, University of Western Australia, Perth 6009, Australia.; ^7^ Downing College, University of Cambridge, Cambridge CB2 1DQ, UK.

## Abstract

Karst landforms provide insights into landscape evolution and paleoclimate but are inherently challenging to date. An ancient interval of particularly intense weathering of Western Australian Pleistocene aeolianites is recorded in a spectacular pinnacle karst landscape with associated ferricrete nodules. (U-Th)/He dating of the ferricrete nodules revealed an age of 102.8 + 10.6/−11.4 thousand years, corresponding to marine isotope stage 5c. The (U-Th)/He age thus directly dates the wettest interglacial period in the region over the last 500 thousand years, which was responsible for the dissolution that formed the pinnacles. The reliability of the ferricrete (U-Th)/He age is supported by bounding optically stimulated luminescence and U-Th dates on associated aeolianites and carbonate precipitates, respectively. A (U-Th)/He approach is globally applicable to aeolianites with associated ferricretes, allowing more accurate dating of the environmental changes affecting these lithologies, and temporally constraining rapid Pleistocene climatic oscillations to better contextualize the associated evolution of the biosphere.

## Teaser

A geochronological approach to dating karst landforms shows a much wetter Western Australia a hundred thousand years ago.

## INTRODUCTION

Pleistocene climate oscillations have triggered profound environmental and evolutionary changes (*1*, *2*). Environmental responses to climatic forcings can be interpreted from landforms, which can preserve evidence of the specific conditions required for their formation. Karst landscapes, which comprise 15% of the present ice-free surface of Earth (*3*), contain landforms that are especially susceptible to modification by climate change. This is because the processes that form karst landforms (i.e., dissolution of susceptible lithologies—limestone, dolomite, and gypsum) are strongly dependent on climate (*4*, *5*), with dissolution most effective during humid climate phases when there are greater quantities of mobile water. As a result, karst landforms, both surface and subsurface features, have been used to provide insights into climate change and landscape evolution during the Pleistocene and over longer time periods spanning millions of years (*6*–*8*).

To maximize the interpretive potential of karst landforms for paleoclimatic reconstruction, a robust chronology of karstification is essential. This poses a major challenge because, ironically, karst landforms in general are defined by empty (i.e., dissolved) spaces within the rock, such as caves, dolines, karren, and solution pipes. As such, karst epitomizes one of the most profound problems in geology in terms of identifying the temporal significance of gaps in the rock record (*9*). Traditionally, temporally constraining karst episodes has focused on either the host rock or precipitates/deposits overlying karst surfaces, which are older and younger than the time of karstification, respectively. Dating of underground deposits like clastic cave sediment and speleothems can also only provide a minimum age of karstification, because like sediment fills, speleothems form after the draining of the cave in which they precipitated (*5*, *10*–*12*). One of the rare successes in dating karst development was achieved by ^40^Ar/^39^Ar dating of alunite that precipitated during the genesis of hypogene caves (*13*), but this technique is limited to hypogene karst, which forms without any direct genetic relationship to the surface climate (*14*).

Fortunately, it is now possible to date ferricretes, which form on the surface of some karst landscapes as a result of strong weathering and consequent soil formation during periods of extensive karstification (*15*, *16*). Ferricretes occur as ferruginous duricrusts cemented by iron oxides (*17*) and authigenic pisoliths and nodules in nonferruginous sediment (*18*), and often form as a response to environmental changes (*19*). Early attempts to date ferricretes using paleomagnetism (*16*, *20*) and the U-Th technique (*21*, *22*) have commonly yielded results with high uncertainties. Furthermore, many samples are not suitable for the latter method due to a low U-Th content. More recently, (U-Th)/He dating has been demonstrated to yield accurate and sufficiently precise ages on ferricretes extending back through the Cenozoic (*23*–*26*). Consequently, this method holds great potential for unraveling paleoclimatic records but, to date, has not been applied to ferricretes younger than half a million years.

**Fig. 1.**
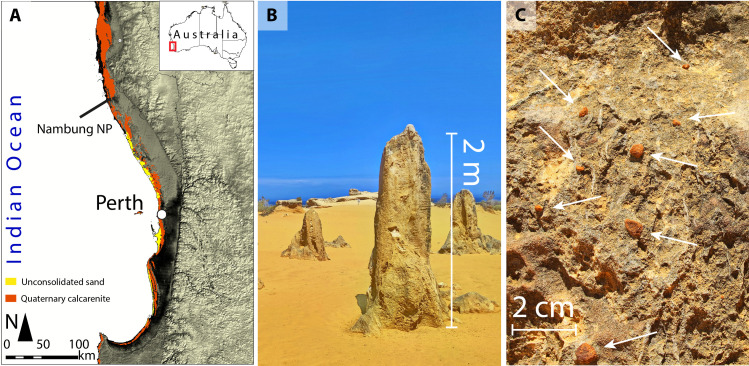
The pinnacles in Nambung National Park, Australia. Locality map of the pinnacles in Nambung National Park, including the distribution of Quaternary calcarenite and Holocene unconsolidated sand (**A**) [based on Geoscience Australia 1:1,000,000 scale, Surface Geology of Australia (digital dataset, 2008); digital elevation model downloaded from the Shuttle Radar Topographic Mission website]. A photograph of pinnacles and stratigraphical sequence in Nambung National Park (**B**). A cluster of ferricrete nodules cemented to the margins of the pinnacles (arrows pointing to nodules) (**C**).

**Fig. 2.**
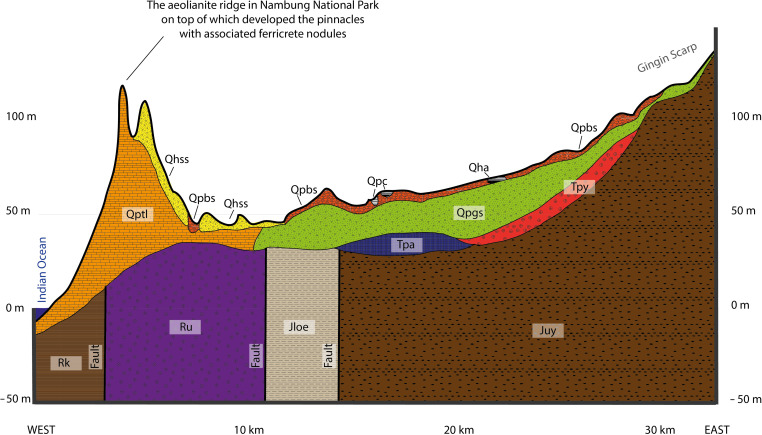
Geological cross section of the coastal dunes, inland plain, and the escarpment. Vertical exaggeration × 100. Qhss, Holocene Safety Bay Sand (loose calcareous sand); Qha, Holocene alluvium; Qptl, Pleistocene Tamala Limestone calcarenite; Qpbs, Pleistocene Bassendean Sand (quartz sand); Qpc, Pleistocene colluvium/sand/laterite; Qpgs, Pleistocene Guildford Formation (sand); Tpy, Pliocene Yoganup Formation; Tpa, Pliocene Ascot Formation; Ru, Triassic Lesueur Sandstone; Rk, Triassic Kockatea Shale; Juy, Jurassic Yarragadee Formation; Jloe, Jurassic Eneabba Member of the Cockleshell Gully Formation [reworked from Kern (*51*)].

Pleistocene climate studies in Australia using karst landscapes have focused on carbonate aeolianites (i.e., aeolian calcarenites or dune limestones) (*27*), particularly along the southern and western coastlines where the world’s most extensive aeolianite deposits occur (*28*) (Fig. 1A). Karstification of the aeolianites, particularly during humid climate phases, has formed a variety of surface and subsurface landforms, including solution pipes, pinnacles, and caves (*29*). Here, we target the world famous pinnacle karst in Nambung National Park (coastal southwestern Western Australia), which consists of thousands of pinnacles up to 5 m high, 2 m wide, and 0.5 to 5 m apart (*30*) (Fig. 1B). The pinnacles are residual features resulting mainly from solution widening and coalescence of vertical solution pipes within the host carbonate aeolianite (*30*). The karst pinnacles are developed in a ridge of aeolianite that runs parallel to the shoreline and lies around 100 m above sea level (Fig. 2). Rounded red-brown ferricrete nodules and pisoliths are widespread over the ridge, occurring cemented on the sides of the pinnacles (Fig. 1C) and scattered around the bases.

Reconnaissance dating of the host rock and postkarstification chemical and clastic deposits by optically stimulated luminescence (OSL) and U-Th methods suggest that the pinnacle-forming karstification episode occurred within the last half million years between marine isotope stage (MIS) 6 and MIS 4, during a period of unusually high effective rainfall (*6*, *31*). Thus, the pinnacle karst in the Nambung National Park may provide evidence of the wettest interglacial period in the Middle-Late Pleistocene. However, published geochronological data for this site, which currently rely on bracketing materials, only indirectly and loosely constrain the age of pinnacle formation. Therefore, there is a need for more precise geochronology that is, ideally, obtained directly on the landform/landscape of interest.

Here, we constrain the age of karstification in Nambung National Park using (U-Th)/He dating of ferricrete nodules cemented on the sides of the pinnacles, thereby dating the time of higher effective rainfall that formed the pinnacles themselves. This provides information on climate changes in southwestern Australia during the Middle-Late Pleistocene and demonstrates the efficacy of the (U-Th)/He geochronology method to date ferricrete material as young as Early Pleistocene.

## RESULTS

The ferricrete analyzed is composed primarily of goethite [identified by x-ray diffraction (XRD); figs. S1 to S4], cementing regolith material composed predominantly of detrital sand to silt grade quartz (16% to 79%) with some feldspar (1% to 2%) as well as clays, consistent with previous studies (*31*). Accessory, finer-grained heavy mineral phases such as rutile, monazite, and zircon are also present in low concentrations (<<1%) dispersed in varying abundance in the different nodules both within the goethite cement and bound within sand-sized clasts (see figs. S1 to S7 and tables S1 and S2 for detailed characterization).

**Fig. 3.**
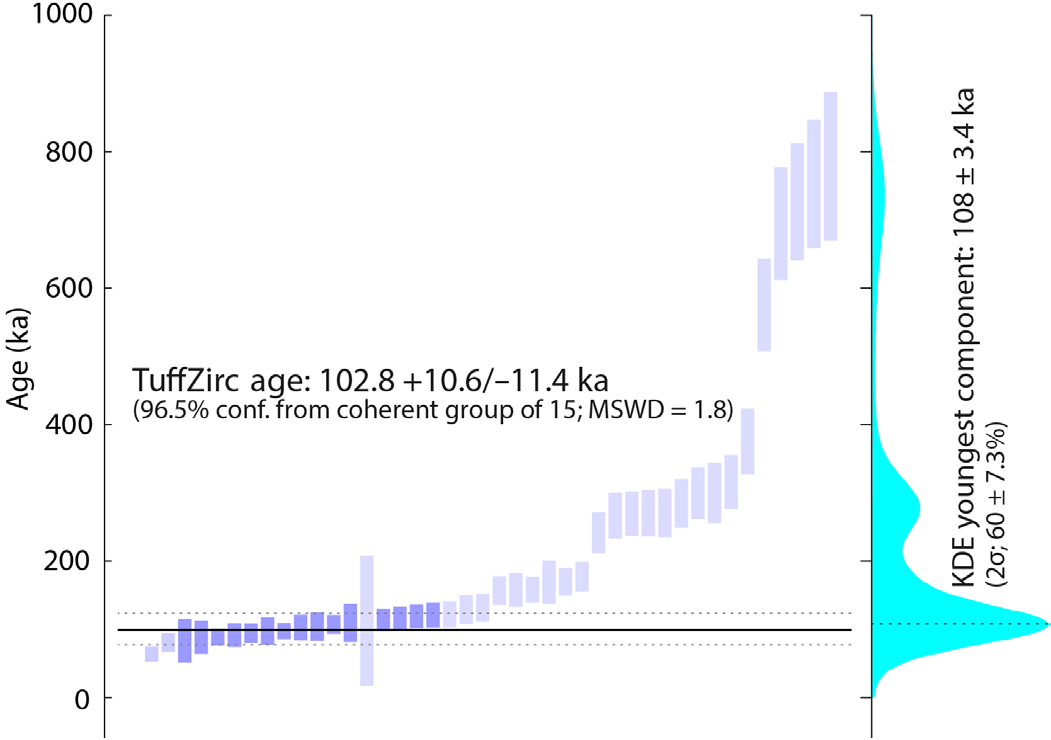
Rank order plot for all (U-Th)/He dates combined, with the youngest statistically coherent age population determined by the TuffZirc and KDE approaches. The *y* axis is limited to <1 Ma for clarity. Note that the difference in uncertainties of the youngest age population determined by the TuffZirc and KDE approach is primarily due to a different treatment of the uncertainties of individual measurements, whereby the KDE approach uses a narrower bandwidth near the modes of the sampling distribution resulting in smaller uncertainties.

Forty-five shards of six ferricrete nodules were dated using the (U-Th)/He method (Table 1, Fig. 3, and fig. S8). The single-shard (U-Th)/He datasets for individual nodules contain some anomalously old outliers and also show substantial scatter as indicated by elevated MSWD values (Table 1). We use the stratigraphic and textural data (*6*, *31*) to infer that all six analyzed ferricrete nodules formed during the same time period (i.e., between MIS 6 and MIS 4). Kernel density estimation (KDE) and the TuffZirc approach, which we used to identify the youngest statistically coherent age population in the entire (U-Th)/He dataset, yield an age mode of 108 ± 3.4 thousand years (ka) (2σ uncertainty; 60 ± 7% of measurements; KDE) and 102.8 + 10.6/−11.4 ka [96.5% confidence interval based on a coherent group of 15 (U-Th)/He dates], respectively (Fig. 3 and fig. S8). Our preference for interpretation purposes is the TuffZirc age (102.8 + 10.6/−11.4 ka) because this approach defines a larger confidence interval, and, given the complexity of the data, the resulting (analytical) uncertainty is geologically more reasonable.

**Table 1.** (U-Th)/He results of individual shards of ferricrete nodules. TAU, total analytical uncertainty; Raw He date, (U-Th)/He date uncorrected for alpha ejection; Ft, alpha ejection correction factor assumed to be 1 given the size of the analyzed shards; Cor. He date, (U-Th)/He date corrected for alpha ejection; Wt. mean ± 95% conf. Int., error weighted mean ± 95% confidence interval.

Sample code	^232^Th ± 1σ	^238^U ± 1σ	^4^He ± 1σ	TAU	Th/U	Raw He date ± 1σ	Ft ± 1σ	Cor. He date ± 1σ
*Shard number*	(ng ± %)	(ng ± %)	(ncc ± %)	(%)		(both in ka)	(± %)	(both in ka)
**Nodule 1**								
***ML1-1***	1.513 ± 2.3	0.168 ± 2.6	0.0108 ± 7.6	7.8	8.95	169.2 ± 13.3	1 ± 5	169.2 ± 15.7
***ML1-2***	2.193 ± 2.3	0.393 ± 2.7	0.0142 ± 6.3	6.5	5.54	128.8 ± 8.4	1 ± 5	128.8 ± 10.6
***ML1-3***	2.545 ± 2.3	0.128 ± 2.6	0.0081 ± 7.8	8.0	19.74	91.5 ± 7.4	1 ± 5	91.5 ± 8.7
***ML1-4***	1.307 ± 2.3	0.200 ± 2.6	0.0356 ± 2.7	3.2	6.48	575.6 ± 18.2	1 ± 5	575.6 ± 34
***ML1-5***	1.203 ± 2.2	0.200 ± 2.6	0.0427 ± 2.7	3.2	5.97	726.6 ± 23	1 ± 5	726.6 ± 43
***ML1-6***	2.684 ± 2.2	0.122 ± 2.6	0.1401 ± 2.2	2.9	21.81	1526.7 ± 44.2	1 ± 5	1526.7 ± 88.2
***ML1-7***	0.267 ± 2.3	0.037 ± 2.8	0.0014 ± 41.8	41.8	7.24	112.8 ± 47.2	1 ± 5	112.8 ± 47.5
		Wt. mean ± 95% conf. Int. (both in ka):						148 ± 150
							MSWD:	105
**Nodule 2**								
***ML3-1***	6.445 ± 2.3	0.141 ± 2.7	0.0575 ± 3.1	3.8	45.32	284.6 ± 10.8	1 ± 5	284.6 ± 17.9
***ML3-2***	12.210 ± 2.3	0.136 ± 2.7	0.0622 ± 2.5	3.3	89.27	169.7 ± 5.7	1 ± 5	169.7 ± 10.2
***ML3-3***	3.488 ± 2.3	0.085 ± 2.7	0.0331 ± 3.3	3.9	40.58	299.7 ± 11.7	1 ± 5	299.7 ± 19
***ML3-4***	16.965 ± 2.3	0.224 ± 2.7	0.0812 ± 2.7	3.4	75.19	158.1 ± 5.4	1 ± 5	158.1 ± 9.6
***ML3-5***	10.866 ± 2.3	0.092 ± 2.7	0.0870 ± 2.4	3.3	117.4	269.8 ± 8.9	1 ± 5	269.8 ± 16.2
***ML3-6***	15.652 ± 2.3	0.217 ± 2.6	0.0842 ± 2.8	3.5	71.67	177.2 ± 6.2	1 ± 5	177.2 ± 10.8
***ML3-7***	9.646 ± 2.3	0.196 ± 2.6	0.6080 ± 1.6	2.7	48.95	2024.8 ± 54.3	1 ± 5	2024.8 ± 114.9
		Wt. mean ± 95% conf. Int. (both in ka):						200 ± 95
							MSWD:	59
**Nodule 3**								
***ML4-1***	1.487 ± 2.2	0.092 ± 2.7	0.0048 ± 12.7	12.9	16.05	88.4 ± 11.4	1 ± 5	88.4 ± 12.2
***ML4-2***	5.477 ± 2.2	0.153 ± 2.7	0.0210 ± 4.8	5.2	35.45	119.3 ± 6.2	1 ± 5	119.3 ± 8.6
***ML4-3***	1.919 ± 2.2	0.070 ± 2.8	0.0066 ± 8.5	8.7	27.38	104.1 ± 9	1 ± 5	104.1 ± 10.4
***ML4-4***	15.388 ± 2.2	0.339 ± 2.5	0.0455 ± 5.2	5.6	45.02	94.4 ± 5.3	1 ± 5	94.4 ± 7.1
***ML4-5***	1.485 ± 2.2	0.207 ± 2.6	0.0510 ± 3.4	3.8	7.11	752.6 ± 28.3	1 ± 5	752.6 ± 47.1
***ML4-6***	5.949 ± 2.2	0.199 ± 2.6	0.0306 ± 4.0	4.4	29.65	157 ± 6.9	1 ± 5	157 ± 10.5
***ML4-7***	2.844 ± 2.2	0.228 ± 2.6	0.0144 ± 5.6	5.9	12.40	131.8 ± 7.8	1 ± 5	131.8 ± 10.2
		Wt. mean ± 95% conf. Int. (both in ka):						118 ± 56
							MSWD:	36
**Nodule 4**								
***ML5-1***	4.492 ± 2.1	0.154 ± 2.5	0.0399 ± 3.8	4.2	28.93	270.5 ± 11.4	1 ± 5	270.5 ± 17.7
***ML5-2***	2.591 ± 2.1	0.178 ± 2.5	0.0151 ± 5.7	6.0	14.43	157.6 ± 9.4	1 ± 5	157.6 ± 12.3
***ML5-3***	4.442 ± 2.1	0.262 ± 2.5	0.0102 ± 6.6	6.8	16.84	63.8 ± 4.4	1 ± 5	63.8 ± 5.4
***ML5-4***	2.531 ± 2.1	0.262 ± 2.5	0.4512 ± 3.0	3.4	9.60	4323.2 ± 147.9	1 ± 5	4323.2 ± 261.9
***ML5-5***	2.332 ± 2.2	0.165 ± 2.6	0.0089 ± 7.4	7.6	14.05	102.8 ± 7.8	1 ± 5	102.8 ± 9.4
***ML5-6***	2.582 ± 2.1	0.361 ± 2.5	0.0143 ± 5.2	5.5	7.10	121 ± 6.6	1 ± 5	121 ± 9
***ML5-7***	2.909 ± 2.2	0.412 ± 2.5	0.0108 ± 6.5	6.7	7.01	80.9 ± 5.5	1 ± 5	80.9 ± 6.8
***ML5-8***	7.844 ± 2.1	0.607 ± 2.5	0.0944 ± 3.3	3.7	12.83	316.1 ± 11.8	1 ± 5	316.1 ± 19.7
		Wt. mean ± 95% conf. Int. (both in ka):						102 ± 69
							MSWD:	79
**Nodule 5**								
***ML6-1***	22.860 ± 2.2	0.518 ± 2.6	0.1734 ± 3.1	3.7	43.83	241.5 ± 8.9	1 ± 5	241.5 ± 15
***ML6-2***	25.917 ± 2.2	0.305 ± 2.5	0.0837 ± 3.3	3.9	84.23	107.3 ± 4.2	1 ± 5	107.2 ± 6.8
***ML6-3***	8.654 ± 2.1	0.126 ± 2.6	0.0234 ± 4.4	4.8	68.09	88.7 ± 4.3	1 ± 5	88.7 ± 6.1
***ML6-4***	8.730 ± 2.2	0.267 ± 2.5	0.0754 ± 3.3	3.8	32.46	266.6 ± 10.2	1 ± 5	266.6 ± 16.8
***ML6-5***	15.939 ± 2.1	0.551 ± 2.5	0.1416 ± 3.3	3.8	28.72	270.4 ± 10.2	1 ± 5	270.4 ± 16.9
***ML6-6***	12.371 ± 2.1	0.377 ± 2.5	0.1503 ± 3.5	4.0	32.61	375.4 ± 14.9	1 ± 5	375.4 ± 24
***ML6-7***	24.274 ± 2.2	0.543 ± 2.6	0.5932 ± 4.5	4.9	44.38	778.8 ± 38.2	1 ± 5	778.7 ± 54.5
		Wt. mean ± 95% conf. Int. (both in ka):						139 ± 89
							MSWD:	81
**Nodule 6**								
***ML7-1***	1.101 ± 2.5	0.017 ± 2.8	0.0028 ± 18.3	18.5	65.66	83.2 ± 15.4	1 ± 5	83.2 ± 15.9
***ML7-2***	2.569 ± 2.5	0.049 ± 2.8	0.0553 ± 2.3	3.2	52.10	694.6 ± 22.4	1 ± 5	694.6 ± 41.3
***ML7-3***	2.529 ± 2.6	0.129 ± 2.9	0.0100 ± 4.6	5.1	19.46	113.7 ± 5.8	1 ± 5	113.7 ± 8.1
***ML7-4***	1.779 ± 2.5	0.255 ± 2.8	0.0100 ± 5.6	5.9	6.91	122.2 ± 7.2	1 ± 5	122.2 ± 9.4
***ML7-5***	2.315 ± 2.5	0.431 ± 2.8	0.0116 ± 2.9	3.4	5.33	97.5 ± 3.3	1 ± 5	97.5 ± 5.9
***ML7-6***	1.191 ± 2.5	0.085 ± 2.8	0.0049 ± 11.6	11.8	13.92	109.7 ± 12.9	1 ± 5	109.7 ± 14
***ML7-7***	1.614 ± 2.5	0.052 ± 2.9	0.0158 ± 4.9	5.4	30.53	300.1 ± 16.3	1 ± 5	300.1 ± 22.2
***ML7-8***	1.800 ± 2.5	0.230 ± 2.8	0.0078 ± 8.7	8.9	7.78	97.5 ± 8.6	1 ± 5	97.5 ± 9.9
***ML7-9***	3.802 ± 2.5	0.388 ± 2.8	0.0183 ± 4.4	4.8	9.74	117 ± 5.7	1 ± 5	117 ± 8.1
		Wt. mean ± 95% conf. Int. (both in ka):						115 ± 45
							MSWD:	35

## DISCUSSION

### Ferricrete age and relationship to karstification

The regolith is interpreted to have developed contemporaneously with pinnacle formation during a major period of karst dissolution. The ferricrete nodules and pisoliths formed within the regolith due to the migration and accumulation of iron oxides, which cemented the detrital material. Ferricrete formation was most likely favored by the low permeability of the regolith due to its clay content, so that seeping rainwater became reducing, increasing iron mobility; these conditions are commonly associated with ferricrete formation (*18*).

Following pinnacle and ferricrete development, the large amounts of quartz sand released by extensive dissolution of the limestone to form the pinnacles was (re)deposited throughout the Nambung region. This period of soil/sand erosion exposed the pinnacles, which were originally subsurface features (*30*). At the same time, the ferricrete nodules were released from the regolith and some became cemented around the pinnacle margins (Fig. 1C). The aeolianite ridge on which the pinnacles are formed is bounded on the inland side by a plain ~50 m lower in elevation (Fig. 2); thus, the ferricrete nodules on the ridge are interpreted to be locally formed and not to have been transported from the plateau further inland (where older ferricrete is abundant).

Given the geomorphological, stratigraphical, and textural limitations on inheritance of intact older ferricrete nodules, the anomalously old (U-Th)/He dates and the overdispersion are principally attributed to the presence of U- and/or Th-rich mineral phases (e.g., zircon; see Supplementary Text and figs. S5C, S6, S7, and S9). While no evidence for complex growth phases or cryptic inheritance of goethite was resolvable via electron microscopy in the investigated samples, imaged U- and Th-bearing inclusions that would have been either (i) unresolvable during sample selection (<5 μm median grain size of zircon) or (ii) immediately adjacent to selected pristine samples can readily explain the recorded age dispersion toward older dates. Such mineral inclusions could provide “parentless” helium to the (U-Th)/He system in the analyzed shards, ultimately resulting in age determinations older than the true ages of the iron-bearing precipitate (*32*). Consequently, our interpretation focuses on the youngest (U-Th)/He dates that are the least likely to be affected by the mineral inclusions.

**Fig. 4.**
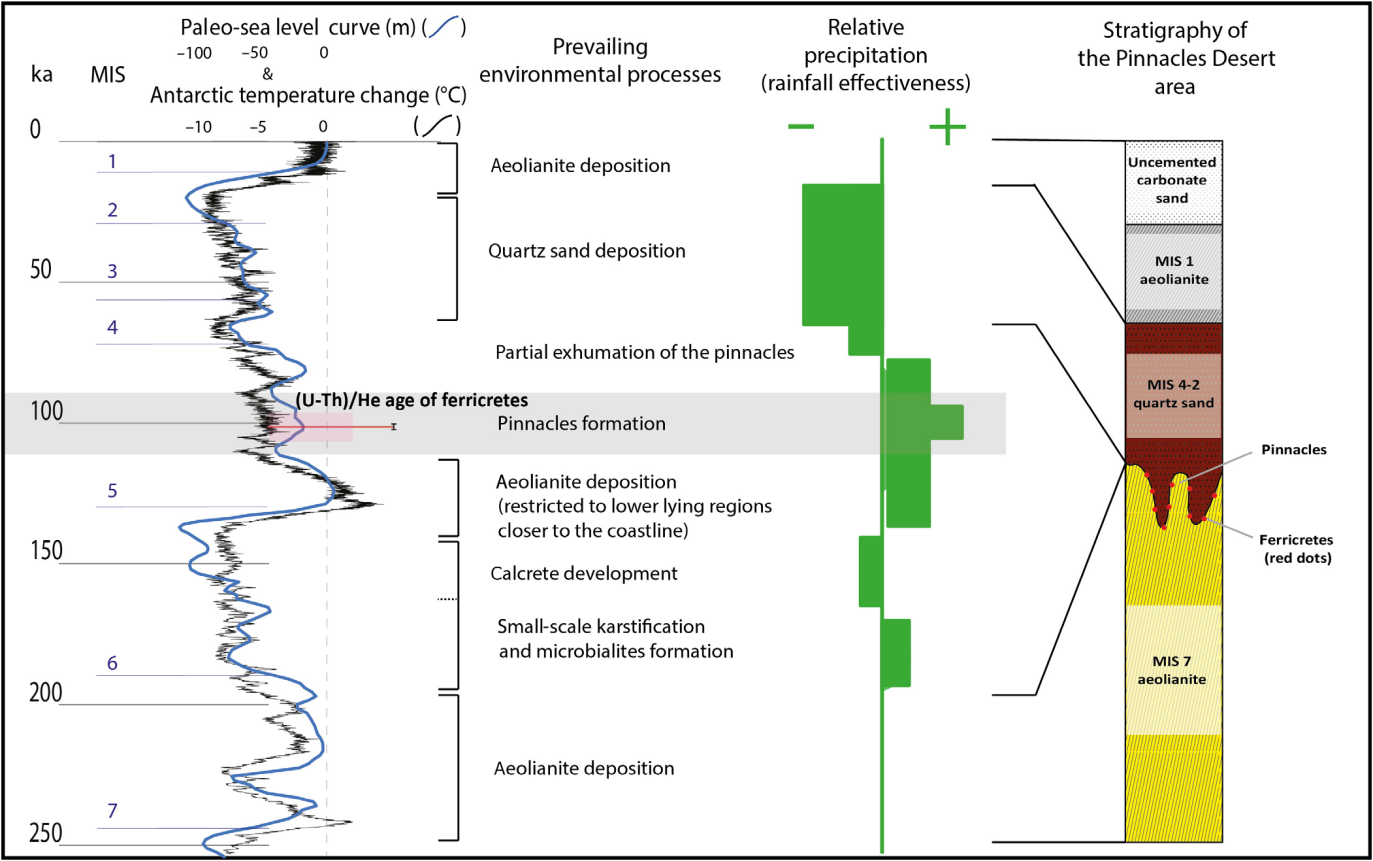
Interpreted stratigraphy and paleoclimate history for the last 250 ka. On the basis of Lipar *et al.* (*6*), updated with the dates of the ferricrete nodules {paleotemperature curve from the European Project for Ice Coring in Antarctica [EPICA, Lemieux-Dudon *et al.* (*52*) and Jouzel *et al.* (*53*)] and sea-level history from Waelbroeck *et al.* (*54*)}.

Because the ferricrete nodules originated during the intense period of karstification that formed the pinnacles, (U-Th)/He dating of the ferricrete provides an accurate date for the karstification event, which, at an age of 102.8 + 10.6/−11.4 ka, occurred during MIS 5c. This age is consistent with the older age of the aeolianite and associated carbonate cements of the pinnacles’ host rock [ca. 210 to 180 ka, MIS 7 and MIS 6; based on OSL and U-Th dating (*6*, *31*)], and the younger age of the quartz sand [ca. 50 to 30 ka, MIS 4 to MIS 2; based on OSL dating (*6*, *31*)] that covers and therefore postdates the pinnacles and ferricrete nodules (Fig. 4).

### Climatic implications of ferricrete formation

Pinnacle karst with ferricrete nodules in Nambung National Park developed only once in a sequence of stacked interglacial aeolianites and glacial soil horizons that extend back to MIS 13 (ca. 500 ka). This ferricrete development is interpreted as an unprecedented period of increased effective rainfall and chemical weathering during MIS 5c, suggesting that this was the wettest interglacial period in the region, when rainfall was probably more efficient than at any other time in the previous 500 ka (Fig. 4). This is in stark contrast to the present Mediterranean climate.

More broadly, deposition of MIS 5 aeolianites in southern Australia was generally restricted to lower elevations, in contrast to more widespread aeolianite deposition during the interglacial periods MIS 11, 9, 7, and 1 (*6*, *30*). This is interpreted to reflect sediment stabilization from more abundant vegetation during a wetter MIS 5, resulting in limited dune migration. The restriction of aeolianite deposition during MIS 5 was not due to weaker wind strength, because this would not have caused more intense karstification. In southeastern Australia, karstification of aeolianites during MIS 5 was less intense than in southwestern Australia, in that there was no pinnacle development (*33*), although both areas were subject to the same period of weathering (one interglacial-glacial cycle). Once the width and distribution of solution pipes become balanced with the rainfall and denudation rate in an area, their subsequent growth is mostly in depth (*34*). Thus, it appears that the solution pipes in southeastern Australia increased in depth rather than diameter as they adjusted to a relatively low rainfall effectiveness. This implies that the effective rainfall in southwestern Australia was higher, sufficient to increase the width of the solution pipes enough to form pinnacles (*6*).

Given recent work demonstrating antiphase MIS 5 peak precipitation in southeastern and southwestern Australia (*35*), recognition of unprecedently wet MIS 5c in the Nambung study region via (U-Th)/He dating of ferricrete nodules herein may be interpreted as a regional continental signal of atmospheric forcings. Such chronologically constrained interpretations provide additional input to detailed regional climate models that are critical to resolving patterns beyond the resolution of coarser paleoclimate data (*36*). In particular, the inferred high rainfall during MIS 5c can be related to different forcing combinations. Western frontal systems bring much of the rainfall to southern Australia, and during MIS 5c the northern parts of southwestern Australia may have experienced the more southerly reach of these systems, along with a greater intensity and frequency of tropical cyclones, which are likely to be the main weather system responsible for pinnacle formation. An alternative driver of the increased rainfall is the Leeuwin Current; this southerly flowing warm ocean current has the effect of raising sea surface temperatures off the southwest Australian coast, with a consequent positive impact on rainfall along the western margin of the continent (*37*). Prolonged higher sea surface temperatures could have also coincided with a more southerly flow of atmospheric rivers over the southeastern Indian Ocean (*38*), adding a further explanation for a much higher rainfall regime during MIS 5c in southwestern Australia.

### Global implications

The Western Australian coastline hosts the largest tract of Quaternary aeolianite in the world, but this lithology occurs along shorelines globally, including the Mediterranean, Middle East, southern and southeastern African coast and associated islands, Indian subcontinent, southeastern Asia, western North America, Caribbean, Bermuda, and some of the Pacific islands (*28*). These aeolianites have been used to investigate dynamic coastal environments and paleoclimates (*28*, *39*–*41*), as well as Late Pleistocene terrestrial biota (*42*) and hominin ecology (*43*). The noncarbonate constituents of the aeolianites (*44*–*46*) can produce ferricrete when strongly weathered, so that detailed stratigraphic and sedimentological investigations of the aeolianites, together with U-Th/He dating of interbedded ferricretes, are likely to be a globally applicable approach. Even where high clastic component concentrations and associated high-eU mineral inclusions complicate (U-Th)/He age populations, meaningful age interpretation of ferruginous precipitation is possible through scanning electron microscopy–based (herein) or microCT (*47*) sample characterization. This will allow accurate dating of the climatic shifts modifying a range of landscapes, providing a more refined timeline of past environmental changes and context for patterns of evolution and biodiversity.

## MATERIALS AND METHODS

### Field work

Detailed field mapping of the characteristics and extent of the ferricrete nodules was performed in Nambung National Park, Western Australia. The present exposure of the pinnacles in this area (elsewhere usually buried in younger quartz sand) offered the opportunity to discretely sample the nodules without an impact on the landscape.

### Petrography

Thin sections with standard thicknesses of 30 μm were prepared for five ferricrete nodules at the Faculty of Natural Sciences and Engineering of the University of Ljubljana and examined using a polarizing microscope.

### X-ray diffraction

The mineralogy was determined using XRD on a Philips x-ray diffractometer (PW 3710) on unoriented powder mounts at the Faculty of Natural Sciences and Engineering of the University of Ljubljana. Qualitative and semiquantitative estimations were based on peak intensity measurement of x-ray patterns (Cu Ka/Ni 40 kV, 30 mA, 2 to 70° 2θ, rate of speed of 3.4θ/min) using the X’Pert HighScore software.

### X-ray fluorescence

The concentration of chemical elements was determined by x-ray fluorescence. The samples were ground into fine powder and pressed into pellets. The Niton XL3t analyzer was used at the University of Ljubljana.

### Scanning electron microscopy

For microstructure and chemical composition, the polished slabs of five ferricrete samples were made and examined using a Thermo Fisher Scientific Quattro S scanning electron microscope equipped with the Oxford Instruments UltimMax 65 EDXS detector at the University of Ljubljana. The instrument was operated in high-vacuum conditions at an accelerating voltage of 15 kV, a 10-nA beam current, and a constant analytical working distance of 10 mm. The instrument was calibrated with pure silicon and cobalt standards for light and heavy elements, respectively. The error in weight % concentration at the 2σ level ranged from 0.05 to 0.25 for light elements and 0.30 to 0.40 for heavy elements. The EDS spectra were acquired over a period of 60 s. Before starting the analyses, the samples were coated with a thin film of amorphous carbon to ensure the electrical conductivity of the sample and prevent charge buildup.

Additional fragments from the dated nodules were mounted in a 25-mm-diameter epoxy resin disc and polished to a 1-μm finish. The mount was coated with 20 μm of carbon and dot-mapped using a TESCAN Integrated Mineral Analyzer (TIMA) under high-vacuum conditions with an accelerating voltage of 25 kV, 5.84 nA beam current, and a constant analytical working distance of 15 mm at the John de Laeter Centre, Curtin University. Backscattered electron images were captured at a 1-μm resolution with EDS captured every 15th step. Backscattered electron image contrast data were integrated with energy-dispersive x-ray spectroscopy to create a mineral map of the samples.

### (U-Th)/He dating

Six ferricrete nodules were selected for (U-Th)/He dating. The nodules were individually crushed in a mortar and the 100- to 300-μm size fraction was sieved off. From this fraction, seven to nine (depending on the size of a nodule) shards of goethite of structurally and compositionally homogeneous appearance were handpicked under a binocular microscope. The shards were then ultra-sonicated in ethanol to remove adherent particles and dried. Selected shards were individually loaded into niobium (Nb) microtubes for (U-Th)/He analysis. (U-Th)/He analyses were carried out at the Western Australia ThermoChronology Hub facility of the John de Laeter Centre (Curtin University, Perth) following the analytical procedures of Danišík *et al.* (*48*). The Nb microtubes containing the goethite shards were loaded into an ultrahigh-vacuum extraction line (Alphachron I) and degassed in a single, 10-min-long heating cycle at ~450°C using a diode laser to circumvent possible changes in the composition of the parent isotopes (*48*). Initial test work before analysis demonstrated that this degassing protocol resulted in the complete outgassing of the samples without the need to “re-extract” the samples. Evolved gas was purified using a “cold finger” cooled with liquid nitrogen and a hot (~350°C) Ti-Zr getter, spiked with 99.9% pure ^3^He and introduced into a Pfeiffer Prisma QMS-200 mass spectrometer, next to a cold Ti-Zr getter. ^4^He/^3^He ratios, corrected for HD and ^3^H by monitoring mass 1, were measured by a Channeltron detector operated in static mode. ^4^He contents were determined by isotope dilution using a ^3^He spike against calibrated ^4^He gas standards, with a blank correction check by heating empty Nb tubes using the same procedure. Following the ^4^He measurements, Nb tubes containing the goethite shards were retrieved from the Alphachron I, spiked with ^230^Th and ^235^U, and dissolved in 200 μl of concentrated HCl in Parr acid digestion vessels heated to 200°C for 24 hours. Each digestion vessel also contained blank and spiked standard solutions. All solutions were analyzed by isotope dilution for ^238^U and ^232^Th on an Agilent 7700 ICP-MS. The total analytical uncertainty (TAU) was calculated as the square root of the He analysis uncertainty and weighted uncertainties of the U and Th measurements (*48*). The raw (U–Th)/He dates were not corrected for alpha ejection (Ft-correction) given the size of sub-sampled goethite specimens; however, an additional uncertainty of 10% was added in quadrature to the final (U-Th)/He dates to honor our limited knowledge about the factors potentially affecting the alpha ejection correction in the investigated samples (e.g., U and Th distribution). The measured (U-Th)/He dates are <1 million years (Ma). However, given that measured Th/U ratios in the samples are rather high (range: 5–117; Table 1), suggesting that most alpha particles were produced by Thorium decay series, no correction for disequilibrium in U-series decay chain was applied to the results.

We note that the dissolution protocol used in this study has been demonstrated to dissolve iron oxides; however, the protocol will not dissolve silicate minerals such as zircon. Mineral mapping by TIMA revealed a complex character of detrital minerals within the ferricrete precipitate including U/Th-rich mineral inclusions (zircon, monazite, rutile, etc.), which would contribute to the total helium budget in the analyzed samples. However, if undissolved for U-Th analysis, the U-Th content of these minerals would be omitted from the measurements of the total budget of parent nuclides. Consequently, there will be an “excess” of radiogenic helium in the analyzed samples and the resulting (U-Th)/He age will be erroneously old. Therefore, in complex geological materials such as secondary iron oxides, which commonly contain fluid and/or U/Th-rich mineral inclusions, younger (U-Th)/He dates in overdispersed datasets are deemed more reliable for geological interpretations.

### Statistical analysis

To determine a meaningful representative age from the measured (U-Th)/He dates, two independent approaches were applied: KDE using the DensityPlotter software package (*49*) and TuffZirc using the Isoplot v.4.15 Excel add-in (*50*). Our motivation was to determine the youngest statistically coherent age component in the combined (U-Th)/He dataset given that the data are overdispersed with some anomalously old (U-Th)/He dates. We assume that the overdispersion stems from the complexity of dated material and the presence of mineral inclusions as documented by electron microscopy.

## Supplementary Materials

### This PDF file includes:

Supplementary Text Figs. S1 to S9 Tables S1 and S2 References
