# Supplementary material for: Ironing out complexities in karst chronology: (U-Th)/He ferricrete ages reveal wet MIS 5c
Source: Sci Adv. 2024 Oct 2;10(40):eadp0414. doi: 10.1126/sciadv.adp0414 (PMC11446275; doi:10.1126/sciadv.adp0414)
Supplement: Supplementary file 1 — Supplementary Text Figs. S1 to S9 Tables S1 and S2 References [file sciadv.adp0414_sm.pdf]

Science Advances
AAAS

Supplementary Materials for

Ironing out complexities in karst chronology: (U-Th)/He ferricrete ages reveal wet MIS 5c

Matej Lipar *et al.*

Corresponding author: Matej Lipar, matej.lipar@zrc-sazu.si

Sci. Adv. **10**, eadp0414 (2024)
DOI: 10.1126/sciadv.adp0414

This PDF file includes:

Supplementary Text
Figs. S1 to S9
Tables S1 and S2
References

Supplementary Text

Nodule composition and influence on age results

Nodules 1-6 yielded variably dispersed (U-Th)/He dates. The dispersion is interpreted here to principally represent the incorporation of "parentless" He within the shards. No conclusive detrital goethite/hematite grains were identified during electron microscopy characterization that could have represented older growth phases. Notable quantities of accessory U- and Th-bearing minerals have been identified in all nodules examined (figs. S5-S7). In particular, many of the zircon grains are very fine, ranging from 1.1 to 53.7 µm (median 4.7 µm) area-equivalent-circle diameter, making them very challenging to identify during selection screening prior to dating. All U- and Th- bearing mineral grains would have been capable of implanting excess He in the measured shards where they were originally directly adjacent, while included zircon grains in any analysed shard would have contributed He but neither U nor Th due to the inability of the HCl based digestion protocol employed herein to completely digest the zircon material. Consequently, resulting (U-Th)/He dates would appear older than the true age of the induration. In support of this interpretation is the observation that nodules that yielded higher proportions of shards that defined consistent young ages, were also those with lower detrital proportions and larger, more pristine areas of ferruginous cementation (figs. S7 and S9). Nodules that yielded more dispersed and older ages were those with more limited cementation and higher relative proportions of detrital grains, making it more challenging to select pristine material that would not have been impacted by U- and Th- bearing minerals.

We also note that one shard yielded an anomalously young age of 63.8 ± 10.8 ka (2σ). We consider this age to be an "outlier" potentially stemming from an incomplete gas extraction caused by misalignment of the sample in laser chamber. No textural evidence was observed for younger induration growth events. Therefore, this age is excluded from geological interpretations. However, we acknowledge that anomalously young (U-Th)/He dates may also result from diffusive loss of Helium at (near)surface conditions. A potential way of testing this possibility would be $^4He/^3He$ thermochronometry *(55)*, which could allow reconstruction of time-temperature trajectories and correction of (U-Th)/He ages for partial diffusive loss *(56, 57)*.

Regardless of any cryptic ferruginous inheritance, younger growth events, or potential diffusive loss at Earth-surface conditions, the coherency and dominance of the young, ca. 103 ka age component supports a period of substantial ferruginisation and the interpretations herein of intense weathering, rainfall and karstification at this time.

Fig. S1.

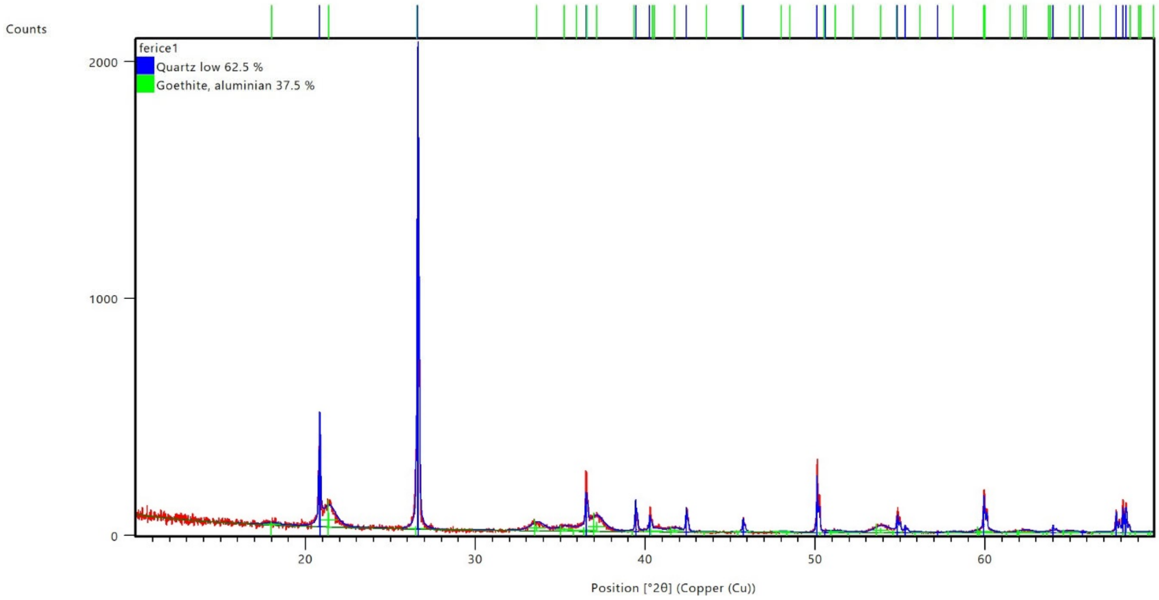

Mineralogy of ferricrete nodule 1 determined by XRD method.

Fig. S2.

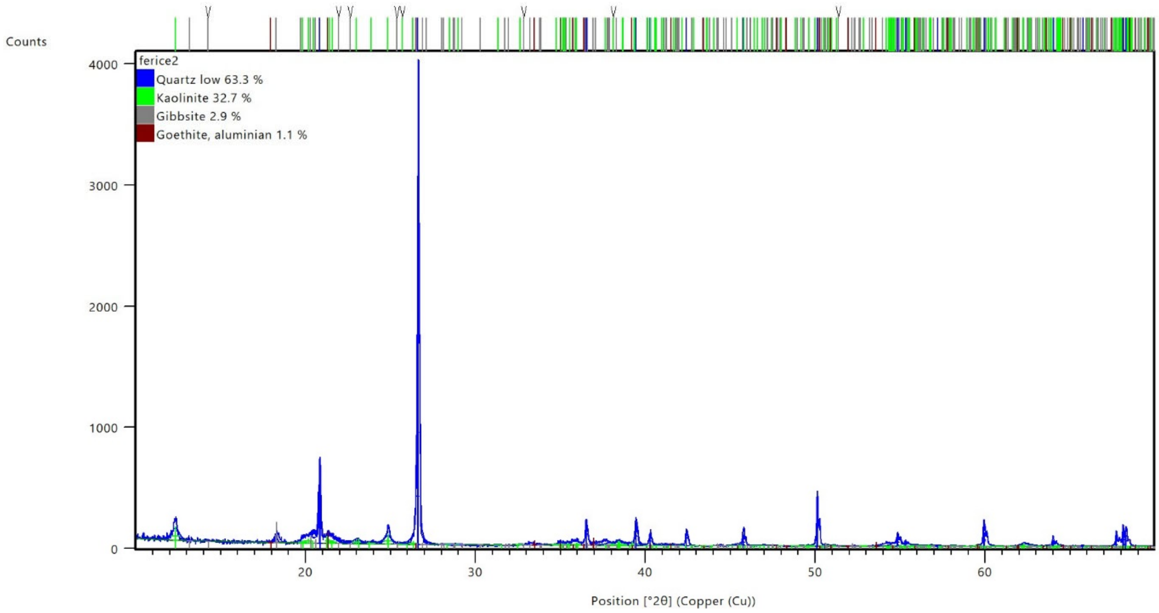

Mineralogy of ferricrete nodule 2 determined by XRD method.

Fig. S3.

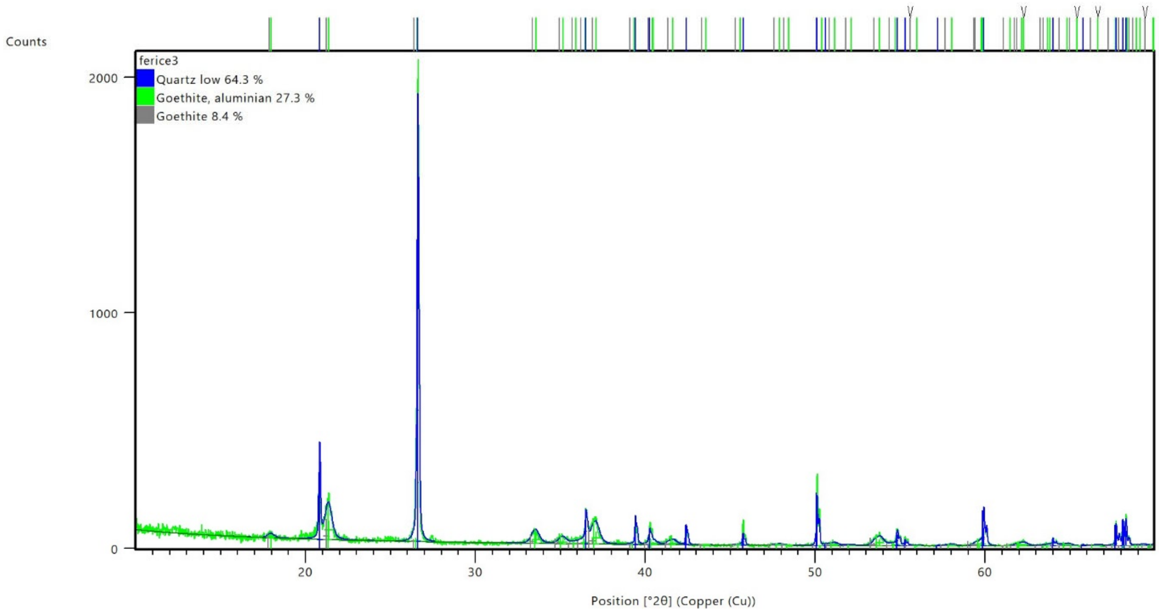

Mineralogy of ferricrete nodule 3 determined by XRD method.

Fig. S4.

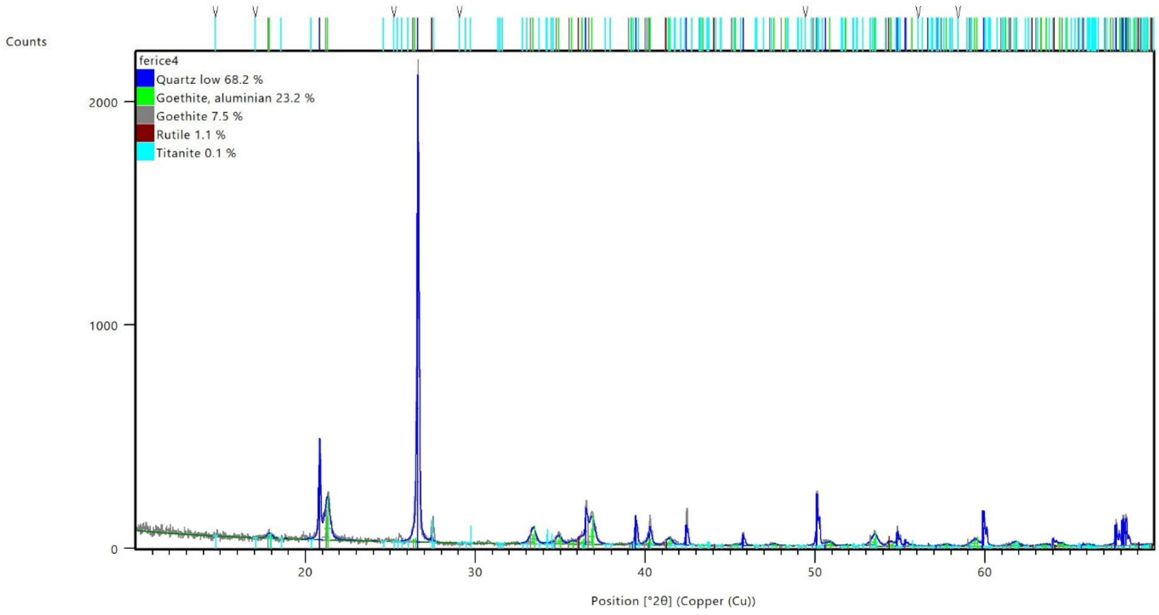

Mineralogy of ferricrete nodule 4 determined by XRD method.

Fig. S5.

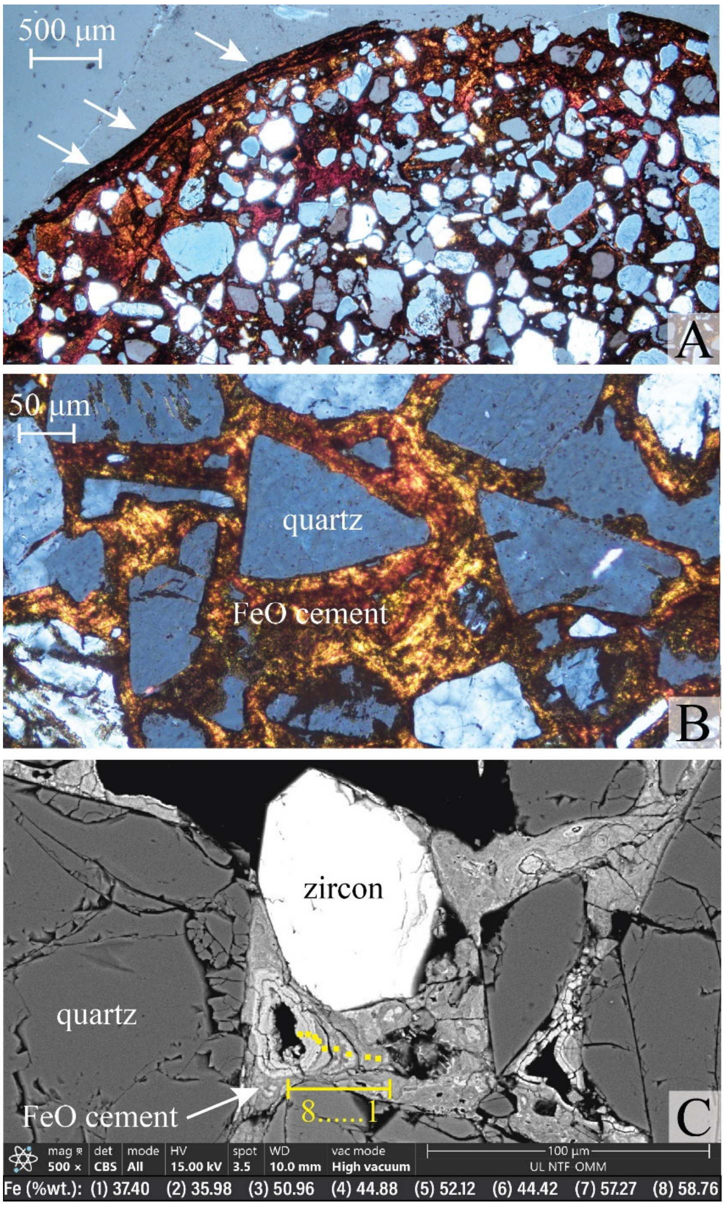

Microphotographs in cross polarised light (A, B) and electron micrograph (C) of a ferricrete nodule. (A) Ferruginous crust (arrows) around the nodule. **(B)** A detail of FeO cement, filling the pores between the grains. **(C)** Elementary X-ray analysis of FeO cement showing larger Fe content in the innermost layers – see SEM X-Ray scan data in Table S2.

Fig. S6

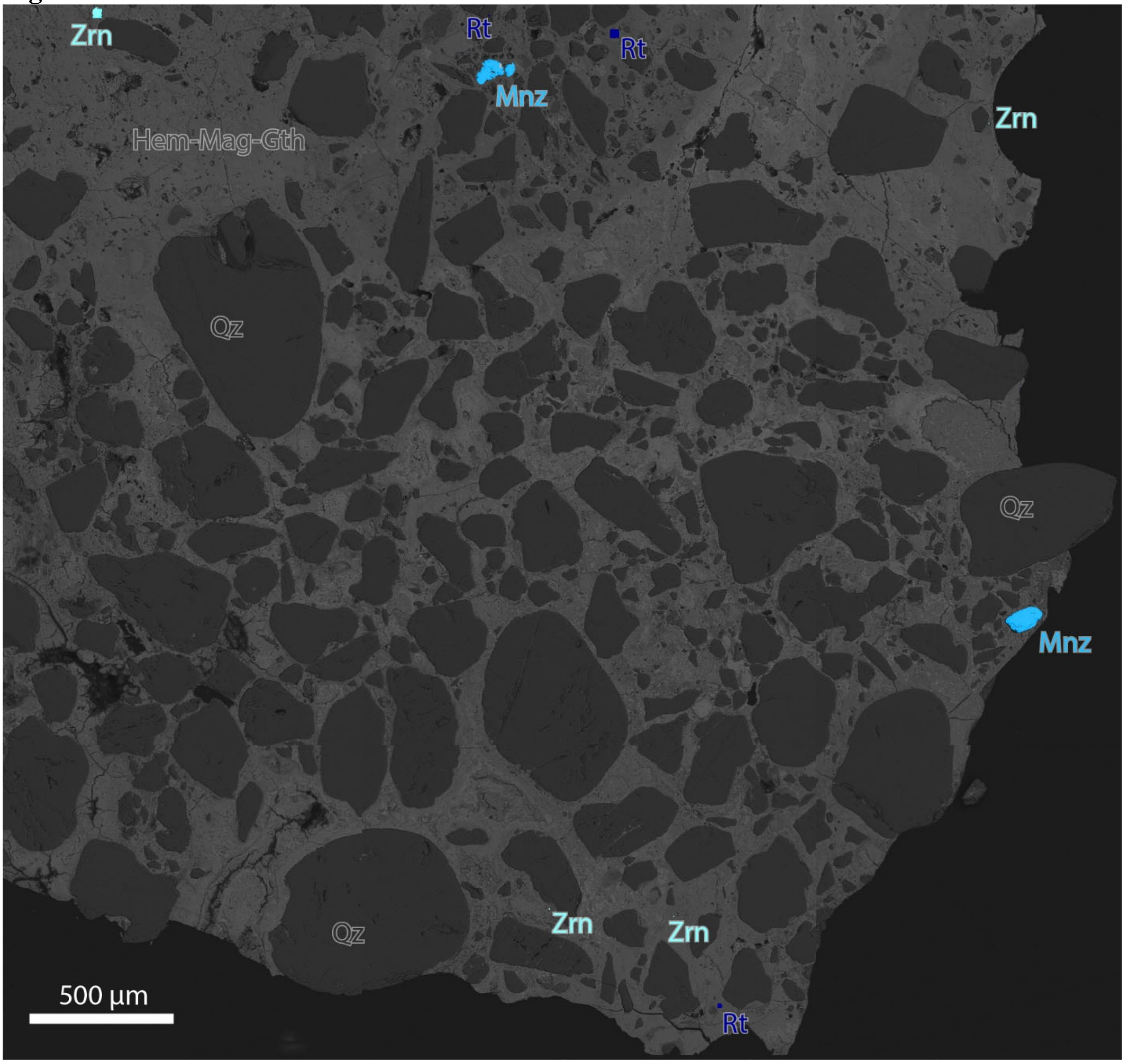

Backscattered electron image of part of nodule N5 showing a dominance of iron induration (characterised as hematite-magnetite-goethite (Hem-Mag-Gth) via energy dispersive X-ray spectroscopy) cementing detrital rounded quartz (Qtz), as well as relevant accessory quantities of variably fine-grained detrital high eU minerals such as monazite (Mnz), zircon (Zrn) and rutile (Rt) that could have contributed to parentless He measured within apparently older shards.

Fig. S7

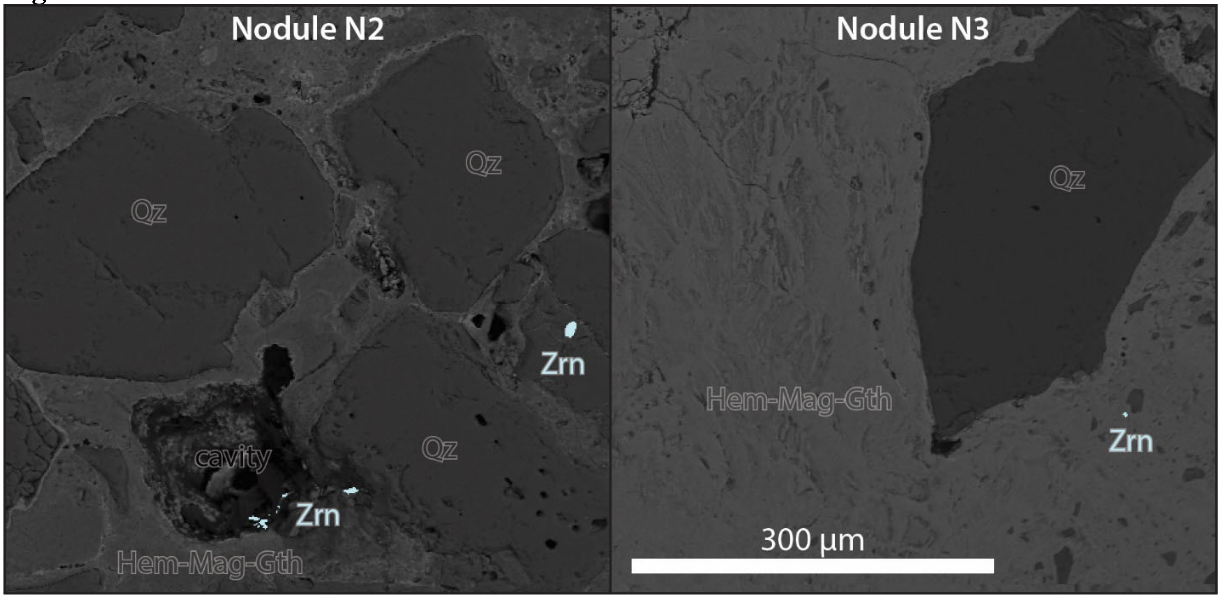

Backscattered electron image with zircon overlay in light blue for representative portions of nodule N2 (dispersed age results) and nodule N3 (relatively consistent young dates). Energy dispersive X-ray spectroscopy characterised minerals labelled as hematite-magnetite-goethite (Hem-Mag-Gth) cementing detrital quartz (Qtz), as well as zircon (Zrn). Note the differences between the nodules in terms of the concentration of detrital minerals in N2 with smaller volumes of intervening iron-bearing cement and the large pristine sections of cement in N3.

Fig. S8.

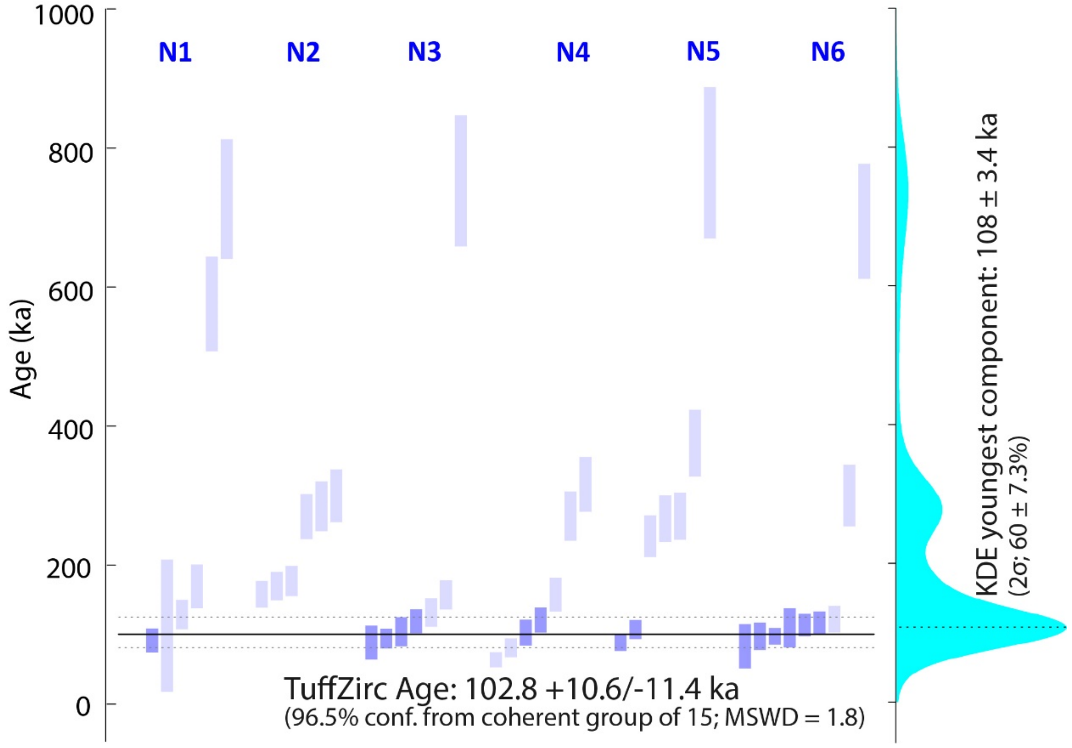

Rank order plots of (U-Th)/He dates for individual ferricrete samples.

Fig. S9

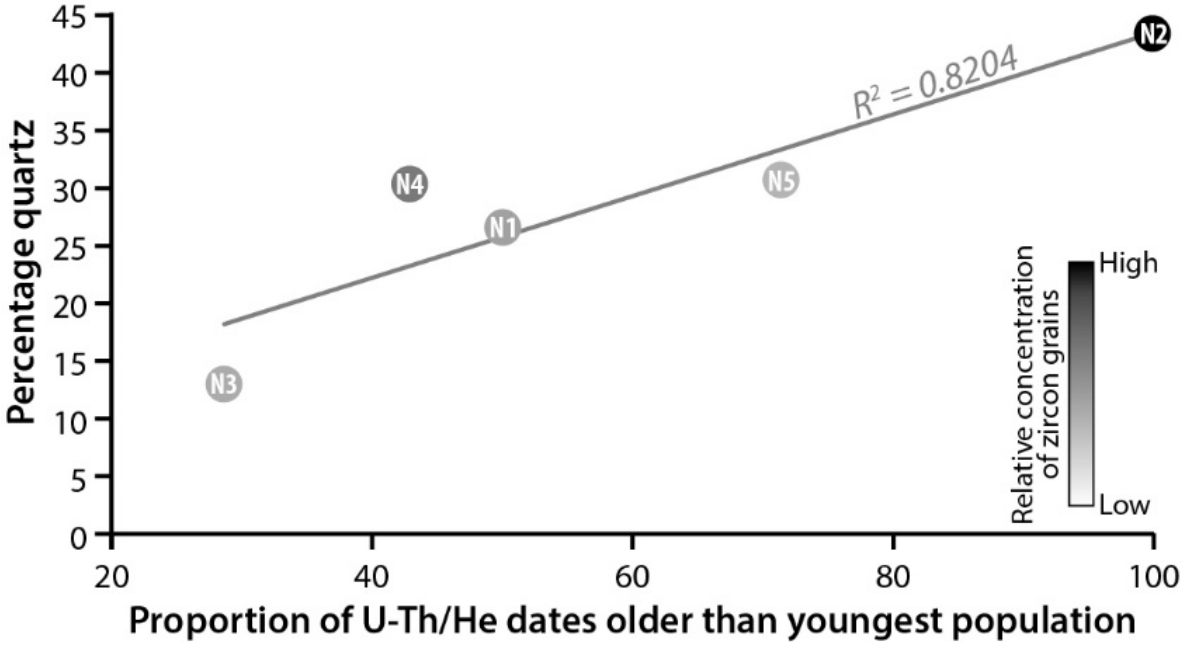

Relationship between the proportion of measurements older than the youngest population and the volume of detrital material (using quartz as a proxy). Note the increasing apparent (on imaged surface) relative abundance of zircon grains with increasing quartz too. Nodule numbers provided within the data points.

Table S1.

**Sample**	SiO_2	Al_2O_3	Fe_2O_3	MgO	CaO	K_2O	TiO_2	P_2O_5	MnO	Si	Al	Fe	Mg
	[%]	[%]	[%]	[%]	[%]	[%]	[%]	[%]	[%]	[%]	[%]	[%]	[%]
**Ferricrete 1**	52.09	6.67	26.12	-	0.18	0.07	0.17	-	-	24.35	3.53	18.27	-
**Ferricrete 2**	53.06	23.51	8.16	-	0.12	0.03	0.81	-	-	24.80	12.44	5.71	-
**Ferricrete 3**	45.66	6.43	25.51	-	0.17	0.39	0.38	-	-	21.34	3.40	17.84	-
**Ferricrete 4**	58.48	5.14	24.60	-	0.13	1.50	0.29	-	0.01	27.33	2.72	17.20	-
	Ca	K	Ti	P	S	Cl	Mn	Cr	As	Ba	Cu	Mo	Nb
	[%]	[%]	[%]	[%]	[%]	[%]	[%]	[mg/kg]	[mg/kg]	[mg/kg]	[mg/kg]	[mg/kg]	[mg/kg]
**Ferricrete 1**	0.13	0.06	0.10	-	-	-	-	351	178	300	22	9	10
**Ferricrete 2**	0.08	0.02	0.49	-	-	-	-	350	23	42	15	13	16
**Ferricrete 3**	0.12	0.33	0.23	-	-	-	-	330	113	180	17	14	7
**Ferricrete 4**	0.09	1.25	0.17	-	-	-	0.01	453	334	726	15	8	8
	Pb	Rb	Sr	Zn	Zr	Ag	Au	Bi	Cd	Co	Ni	Pd	Sb
	[mg/kg]	[mg/kg]	[mg/kg]	[mg/kg]	[mg/kg]	[mg/kg]	[mg/kg]	[mg/kg]	[mg/kg]	[mg/kg]	[mg/kg]	[mg/kg]	[mg/kg]
**Ferricrete 1**	44	4	13	-	169	-	-	-	-	-	122	-	-
**Ferricrete 2**	18	2	7	-	378	-	-	-	-	-	32	-	-
**Ferricrete 3**	59	14	16	-	216	-	-	-	-	-	57	-	-
**Ferricrete 4**	64	41	34	-	187	6	-	-	-	-	86	-	-
	Se	Sn	Re	Ta	Hf	W	Hg	Th	U	V	Y	Bal	
	[mg/kg]	[mg/kg]	[mg/kg]	[mg/kg]	[mg/kg]	[mg/kg]	[mg/kg]	[mg/kg]	[mg/kg]	[mg/kg]	[mg/kg]	[%]	
**Ferricrete 1**	3	85	-	-	-	-	-	-	-	302	28	51.96	
**Ferricrete 2**	-	60	-	-	-	-	-	50	-	208	6	52.79	
**Ferricrete 3**	-	106	-	-	-	-	-	-	-	254	25	55.21	
**Ferricrete 4**	-	50	-	-	-	-	-	5	-	553	42	49.91	

Geochemistry of four ferricrete nodules determined by XRF method.

Table S2.

Spectrum Label	FeO_1	FeO_2	FeO_3	FeO_4	FeO_5	FeO_6	FeO_7	FeO_8
Fe	37.40	35.98	50.96	44.88	52.12	44.42	57.27	58.76
O	41.81	43.16	39.70	40.33	35.85	40.34	37.98	36.86
Al	11.39	11.51	5.92	8.11	6.89	8.38	3.30	3.00
Si	9.41	9.36	2.73	6.68	5.13	6.87	1.45	1.38
Ca			0.28					
Cr			0.41					
Total	100.00	100.00	100.00	100.00	100.00	100.00	100.00	100.00

SEM X-ray geochemistry of the iron cement (see fig. S5(C) for sampling location).

REFERENCES AND NOTES

1. F. V. Seersholm, D. J. Werndly, A. Grealy, T. Johnson, E. M. Keenan Early, E. L. Lundelius Jr., B. Winsborough, G. E. Farr, R. Toomey, A. J. Hansen, B. Shapiro, M. R. Waters, G. McDonald, A. Linderholm, T. W. Stafford Jr., M. Bunce, Rapid range shifts and megafaunal extinctions associated with late Pleistocene climate change. *Nat. Commun.* **11**, 2770 (2020).

2. A. Timmermann, K.-S. Yun, P. Raia, J. Ruan, A. Mondanaro, E. Zeller, C. Zollikofer, M. P. de Leon, D. Lemmon, M. Willeit, A. Ganopolski, Climate effects on archaic human habitats and species successions. *Nature* **604**, 495–501 (2022).

3. N. Goldscheider, Z. Chen, A. S. Auler, M. Bakalowicz, S. Broda, D. Drew, J. Hartmann, G. Jiang, N. Moosdorf, Z. Stevanovic, G. Veni, Global distribution of carbonate rocks and karst water resources. *Hydrgeol. J.* **28**, 1661–1677 (2020).

4. R. Lastennet, J. Mudry, Role of karstification and rainfall in the behavior of a heterogeneous karst system. *Environ. Geol.* **32**, 114–123 (1997).

5. J. de Waele, F. Gutiérrez, *Karst Hydrogeology, Geomorphology and Caves* (Wiley-Blackwell, 2022).

6. M. Lipar, J. A. Webb, M. L. Cupper, N. Wang, Aeolianite, calcrete/microbialite and karst in southwestern Australia as indicators of middle to late Quaternary palaeoclimates. *Palaeogeogr. Palaeoclimatol. Palaeoecol.* **470**, 11–29 (2017).

7. P. Audra, A. Bini, F. Gabrovšek, P. Häuselmann, F. Hobléa, P.-Y. Jeannin, J. Kunaver, M. Monbaron, F. Šušteršič, P. Tognini, H. Trimmel, A. Wildberger, Cave and karst evolution in the Alps and their relation to paleoclimate and paleotopography. *Acta Carsologica* **36**, 10.3986/ac.v36i1.208 (2007).

8. J. Mylroie, M. Lace, N. Albury, J. Mylroie, Flank margin caves and the position of mid- to late Pleistocene sea level in the Bahamas. *J. Coast. Res.* **36**, 249–260 (2020).

9. E. A. Barefoot, J. A. Nittrouer, K. M. Straub, Sedimentary processes and the temporal resolution of sedimentary strata. *Geophys. Res. Lett.* **50**, e2023GL103925 (2023).

10. I. J. Fairchild, A. Baker, *Speleothem Science: From Process to Past Environments* (Wiley Blackwell, 2012).

11. A. Tassy, L. Mocochain, O. Bellier, R. Braucher, J. Gattacceca, D. Bourlès, Coupling cosmogenic dating and magnetostratigraphy to constrain the chronological evolution of peri-Mediterranean karsts during the Messinian and the Pliocene: Example of Ardèche valley, Southern France. *Geomorphology* **189**, 81–92 (2013).

12. R. A. L. Osborne, H. Zwingmann, R. E. Pogson, D. M. Colchester, Carboniferous clay deposits from Jenolan Caves, New South Wales: Implications for timing of speleogenesis and regional geology. *Aust. J. Earth Sci.* **53**, 377–405 (2006).

13. V. J. Polyak, W. C. McIntosh, N. Guven, P. Provencio, Age and origin of carlsbad cavern and related caves from $^{40}Ar/^{39}Ar$ of alunite. *Science* **279**, 1919–1922 (1998).

14. A. B. Klimchouk, The Karst paradigm: Changes, trends and perspectives. *Acta Carsologica* **44**, 289–313 (2016).

15. H. O. Beckford, H. Chu, C. Song, C. Chang, H. Ji, Geochemical characteristics and behaviour of elements during weathering and pedogenesis over karst area in Yunnan–Guizhou plateau, southwestern China. *Environ. Earth Sci.* **80**, 1–21 (2021).

16. H. Théveniaut, F. Quesnel, R. Wyns, G. Hugues, Palaeomagnetic dating of the "Borne de Fer" ferricrete (NE France): Lower Cretaceous continental weathering. *Palaeogeogr. Palaeoclimatol. Palaeoecol.* **253**, 271–279 (2007).

17. J. C. Dixon, 4.3 Pedogenesis with respect to geomorphology, in *Treatise on Geomorphology*, J. F. Shroder, Ed. (Academic Press, San Diego, 2013), pp. 27–43.

18. R. R. Anand, Evolution, classification and use of ferruginous regolith materials in gold exploration, Yilgarn Craton, Western Australia. *Geochem. Explor. Environ. Anal.* **1**, 221–236 (2001).

19. H. Zhao, Y. Sun, X. Qiang, Iron oxide characteristics of mid-Miocene red clay deposits on the western Chinese loess plateau and their paleoclimatic implications. *Palaeogeogr. Palaeoclim. Palaeoecol.* **468**, 162–172 (2017).

20. D. Ellerton, T. M. Rittenour, J. Shulmeister, A. P. Roberts, G. Miot da Silva, A. Gontz, P. A. Hesp, P. Moss, N. Patton, T. Santini, K. Welsh, X. Zhao, Fraser Island (K'gari) and initiation of the Great Barrier Reef linked by Middle Pleistocene sea-level change. *Nat. Geosci.* **15**, 1017–1026 (2022).

21. C. D. Woodroffe, E. A. Bryant, D. M. Price, S. A. Short, Quaternary inheritance of coastal landforms, Cobourg Peninsula, Northern Territory. *Aust. Geogr.* **23**, 101–115 (1992).

22. P. C. Augustinus, S. A. Short, H. Heijnis, Uranium/thorium dating of ferricretes from mid- to late Pleistocene glacial sediments, western Tasmania. *Aust. J. Quat. Sci.* **12**, 295–308 (1997).

23. R. R. Anand, M. A. Wells, M. J. Lintern, L. Schoneveld, M. Danišík, W. Salama, R. R. P. Noble, V. Metelka, N. Reid, The (U-Th)/He chronology and geochemistry of ferruginous nodules and pisoliths formed in the paleochannel environments at the Garden well gold deposit, Yilgarn Craton of Western Australia: Implications for landscape evolution and geochemical exploration. *Minerals* **11**, 679 (2021).

24. M. Dröllner, M. Barham, C. L. Kirkland, M. Danišík, J. Bourdet, M. Schulz, M. Aspandiar, Directly dating Plio-Pleistocene climate change in the terrestrial record. *Geophys. Res. Lett.* **50**, e2023GL102928 (2023).

25. B. M. Heller, S. B. Riffel, T. Allard, G. Morin, J.-Y. Roig, R. Couëffé, G. Aertgeerts, A. Derycke, C. Ansart, R. Pinna-Jamme, C. Gautheron, Reading the climate signals hidden in bauxite. *Geochim. Cosmochim. Acta* **323**, 40–73 (2022).

26. T. Allard, C. Gautheron, S. Bressan Riffel, E. Balan, B. F. Soares, R. Pinna-Jamme, A. Derycke, G. Morin, G. T. Bueno, N. do Nascimento, Combined dating of goethites and kaolinites from ferruginous duricrusts. Deciphering the Late Neogene erosion history of Central Amazonia. *Chem. Geol.* **479**, 136–150 (2018).

27. S. J. McLaren, Aeolianite, in *Geochemical Sediments and Landscapes*, D. J. Nash, S. J. McLaren, Eds. (Blackwell Publishing Ltd., 2007), pp. 149–172.

28. B. Brooke, The distribution of carbonate eolianite. *Earth Sci. Rev.* **55**, 135–164 (2001).

29. M. Lipar, S. Q. White, Quaternary coastal dune limestone, in *Australian Caves and Karst Systems*, J. Webb, S. White, G. K. Smith, Eds. (Cave and Karst Systems of the World, Springer Nature, 2023), chap. 15, pp. 219–232.

30. M. Lipar, J. A. Webb, The formation of the pinnacle karst in Pleistocene aeolian calcarenites (Tamala Limestone) in southwestern Australia. *Earth Sci. Rev.* **140**, 182–202 (2015).

31. M. Lipar, J. A. Webb, Middle–late Pleistocene and Holocene chronostratigraphy and climate history of the Tamala Limestone, Cooloongup and Safety Bay Sands, Nambung National Park, southwestern Western Australia. *Aust. J. Earth Sci.* **61**, 1023–1039 (2014).

32. M. Danišík, B. I. McInnes, C. L. Kirkland, B. J. McDonald, N. J. Evans, T. Becker, Seeing is believing: Visualization of He distribution in zircon and implications for thermal history reconstruction on single crystals. *Sci. Adv.* **3**, e1601121 (2017).

33. M. Lipar, J. A. Webb, S. Q. White, K. G. Grimes, The genesis of solution pipes: Evidence from the Middle–Late Pleistocene Bridgewater Formation calcarenite, southeastern Australia. *Geomorphology* **246**, 90–103 (2015).

34. M. Lipar, P. Szymczak, S. Q. White, J. A. Webb, Solution pipes and focused vertical water flow: Geomorphology and modelling. *Earth Sci. Rev.* **218**, 103635 (2021).

35. R. Weij, J. M. K. Sniderman, J. D. Woodhead, J. C. Hellstrom, J. R. Brown, R. N. Drysdale, E. Reed, S. Bourne, J. Gordon, Elevated southern hemisphere moisture availability during glacial periods. *Nature* **626**, 319–326 (2024).

36. F. Feser, B. Rockel, H. von Storch, J. Winterfeldt, M. Zahn, Regional climate models add value to global model data: A review and selected examples. *Bull. Am. Meteorol. Soc.* **92**, 1181–1192 (2011).

37. K.-H. Wyrwoll, B. J. Greenstein, G. Kendrick, The palaeoceanography of the Leeuwin Current: Implications for a future world. *J. R. Soc. West. Aust.* **92**, 37–51 (2009).

38. T. Shinoda, W. Han, L. Zamudio, X. Feng, Influence of atmospheric rivers on the Leeuwin Current system. *Clim. Dyn.* **54**, 4263–4277 (2020).

39. P. Kindler, P. Hearty, Carbonate petrography as an indicator of climate and sea-level changes: New data from Bahamian Quaternary units. *Sedimentology* **43**, 381–399 (1996).

40. J. E. Mylroie, Late Quaternary sea-level position: Evidence from Bahamian carbonate deposition and dissolution cycles. *Quat. Int.* **183**, 61–75 (2008).

41. B. P. Brooke, J. M. Olley, T. Pietsch, P. E. Playford, P. W. Haines, C. V. Murray-Wallace, C. D. Woodroffe, Chronology of Quaternary coastal aeolianite deposition and the drowned shorelines of southwestern Western Australia—A reappraisal. *Quat. Sci. Rev.* **93**, 106–124 (2014).

42. P. J. Hearty, S. L. Olson, Preservation of trace fossils and molds of terrestrial biota by intense storms in mid–last interglacial (MIS 5c) dunes on Bermuda, with a model for development of hydrological conduits. *Palaios* **26**, 394–405 (2011).

43. C. W. Helm, R. T. McCrea, H. C. Cawthra, M. G. Lockley, R. M. Cowling, C. W. Marean, G. H. H. Thesen, T. S. Pigeon, S. Hattingh, A new Pleistocene hominin tracksite from the Cape South Coast, South Africa. *Sci. Rep.* **8**, 3772 (2018).

44. J. J. Fornós, L. B. Clemmensen, L. Gómez-Pujol, A. S. Murray, Late Pleistocene carbonate aeolianites on Mallorca, Western Mediterranean: A luminescence chronology. *Quat. Sci. Rev.* **28**, 2697–2709 (2009).

45. G. Shtienberg, J. K. Dix, J. Roskin, N. Waldmann, R. Bookman, O. M. Bialik, N. Porat, N. Taha, D. Sivan, New perspectives on coastal landscape reconstruction during the late Quaternary: A test case from central Israel. *Palaeogeogr. Palaeoclim. Palaeoecol.* **468**, 503–519 (2017).

46. D. R. Muhs, J. R. Budahn, J. M. Prospero, G. Skipp, S. R. Herwitz, Soil genesis on the island of Bermuda in the Quaternary: The importance of African dust transport and deposition. *J. Geophys. Res.* **117**, F03025 (2012).

47. F. Hofmann, E. H. G. Cooperdock, A. J. West, D. Hildebrandt, K. Strößner, K. A. Farley, Exposure dating of detrital magnetite using ^{3}He enabled by microCT and calibration of the cosmogenic ^{3}He production rate in magnetite. *Geochronology* **3**, 395–414 (2021).

48. M. Danišík, N. J. Evans, E. R. Ramanaidou, B. J. McDonald, C. Mayers, B. I. A. McInnes, (U–Th)/He chronology of the Robe River channel iron deposits, Hamersley Province, Western Australia. *Chem. Geol.* **354**, 150–162 (2013).

49. P. Vermeesch, On the visualisation of detrital age distributions. *Chem. Geol.* **312-313**, 190–194 (2012).

50. K. R. Ludwig, R. Mundil, Extracting reliable U-Pb ages and errors from complex populations of zircons from Phanerozoic tuffs. *Geochim. Cosmochim. Acta* **66**, 461 (2002).

51. A. M. Kern, The geology and hydrogeology of the superficial formations between Cervantes and Lancelin, Western Australia. *Geol. Surv. Prof. Pap.* **34**, 11–36 (1993).

52. B. Lemieux-Dudon, E. Blayo, J.-R. Petit, C. Waelbroeck, A. Svensson, C. Ritz, J.-M. Barnola, B. M. Narcisi, F. Parrenin, Consistent dating for Antarctic and Greenland ice cores. *Quat. Sci. Rev.* **29**, 8–20 (2010).

53. J. Jouzel, V. Masson-Delmotte, O. Cattani, G. Dreyfus, S. Falourd, G. Hoffmann, B. Minster, J. Nouet, J. M. Barnola, J. Chappellaz, H. Fischer, J. C. Gallet, S. Johnsen, M. Leuenberger, L. Loulergue, D. Luethi, H. Oerter, F. Parrenin, G. Raisbeck, D. Raynaud, A. Schilt, J. Schwander, E. Selmo, R. Souchez, R. Spahni, B. Stauffer, J. P. Steffensen, B. Stenni, T. F. Stocker, J. L. Tison, M. Werner, E. W. Wolff, Orbital and millennial Antarctic climate variability over the past 800,000 years. *Science* **317**, 793–796 (2007).

54. C. Waelbroeck, L. Labeyrie, E. Michel, J. C. Duplessy, J. F. McManus, K. Lambeck, E. Balbon, M. Labracherie, Sea-level and deep water temperature changes derived from benthic foraminifera isotopic records. *Quat. Sci. Rev.* **21**, 295–305 (2002).

55. D. L. Shuster, K. A. Farley, $^{4}He/^{3}He$ thermochronometry. *Earth Planet. Sci. Lett.* **217**, 1–17 (2004).

56. P. M. Vasconcelos, J. A. Heim, K. A. Farley, H. Monteiro, K. Waltenberg, $^{40}Ar/^{39}Ar$ and (U–Th)/He–$^{4}He/^{3}He$ geochronology of landscape evolution and channel iron deposit genesis at Lynn Peak. *Geochim. Cosmochim. Acta* **117**, 283–312 (2013).

57. S. H. Scoggin, P. W. Reiners, D. L. Shuster, G. H. Davis, L. A. Ward, J. R. Worthington, P. A. Nickerson, N. S. Evenson, (U-Th)/He and $^{4}He/^{3}He$ thermochronology of secondary oxides in faults and fractures: A regional perspective from Southeastern Arizona. *Geochem. Geophys. Geosyst.* **22**, e2021GC009905 (2021).
